# Oncogenes and the Origins of Leukemias

**DOI:** 10.3390/ijms23042293

**Published:** 2022-02-18

**Authors:** Geoffrey Brown

**Affiliations:** School of Biomedical Sciences, Institute of Clinical Sciences, College of Medical and Dental Sciences, University of Birmingham, Edgbaston, Birmingham B15 2TT, UK; g.brown@bham.ac.uk; Tel.: +44-(0)121-414-4082

**Keywords:** oncogenes, leukemia, stem cells, lineage fate

## Abstract

Self-maintaining hematopoietic stem cells are a cell population that is primarily ‘at risk’ to malignant transformation, and the cell-of-origin for some leukemias. Tissue-specific stem cells replenish the different types of functional cells within a particular tissue to meet the demands of an organism. For hematopoietic stem cells, this flexibility is important to satisfy the changing requirements for a certain type of immune cell, when needed. From studies of the natural history of childhood acute lymphoblastic leukemia, an initial oncogenic and prenatal insult gives rise to a preleukemic clone. At least a second genomic insult is needed that gives rise to a leukemia stem cell: this cell generates a hierarchy of leukemia cells. For some leukemias, there is evidence to support the concept that one of the genomic insults leads to dysregulation of the tissue homeostatic role of hematopoietic stem cells so that the hierarchy of differentiating leukemia cells belongs to just one cell lineage. Restricting the expression of particular oncogenes in transgenic mice to hematopoietic stem and progenitor cells led to different human-like lineage-restricted leukemias. Lineage restriction is seen for human leukemias by virtue of their sub-grouping with regard to a phenotypic relationship to just one cell lineage.

## 1. Introduction

A precise description of the population structure of the hematopoietic cell system is essential to pinpointing the origins of the many different leukemias. The most complex of the interlinked and diverse cell populations in the adult organism is perhaps the blood cell system. In the 1950s, controversy about the nature of hematopoiesis centered on a monophyletic versus a dualistic (or pluralistic) origin of the various types of blood cells. The monophyletic theory, favored by early 20th century morphologists, is that all of the blood cell types arise from a common and multipotent hematopoietic stem cell (HSC). Dualists argued that the different types of cells have distinct progenitors.

A monophyletic origin gained favor in the late 1950s with the identification of a bone marrow cell that is termed a colony-forming unit in spleen (CFU-S). When single cells were transplanted into mice, CFU-S gave rise to a nodule in the spleen containing, at least, erythrocytes, megakaryocytes, granulocytes, and macrophages. Investigators argued that the mouse CFU-S is a stem cell, but the CFU-S compartment does not encompass all stem cells. Work in the mid-1990s revealed that mouse HSCs reside within a much smaller fraction of bone marrow cells lacking lineage markers and that express the Sca-1 antigen and the c-kit receptor for stem cell factor (termed LSK) and that are CD34low/−. A single LSK, CD34low/− was used to reconstitute hematopoiesis long-term in a lethally irradiated mouse. Bone marrow cell LSK, CD34+ cells were co-transplanted to provide short-term radio-protection [1]. A multipotent HSC that self-renews is, therefore, the ‘real’ or ‘primary’ stem cell for hematopoiesis.

Therefore, and for many years, ‘real’ mouse and human HSCs were ring-fenced, and sorted, as a homogenous population of cells, by virtue of their expression of a number of cell surface markers. HSCs are now seen to be a heterogeneous population of cells and include sub-sets with an affiliation to a single lineage, a propensity to develop along a pathway, and that self-renew. Self-renewal is, therefore, no longer a canonical state of stem cells, nor do stem cells inevitably decrease their self-renewal capacity as they age and differentiation proceeds. This review examines this new view to the architecture of hematopoiesis and whether the existence of multipotent and lineage-affiliated cells that self-renew adds complexity to resolving the ‘target’ cell populations for the various leukemias.

## 2. HSCs Are a Heterogeneous Population of Cells

Panels of monoclonal antibodies to cell surface proteins greatly assisted the description of HSCs. Human HSCs and their immediate offspring are enriched in a population of cells that is LSK CD34+, CD38−. Mouse HSCs have been purified to a much greater extent, and their phenotype is described in various ways. For example, HSCs that reconstitute an irradiated mouse long-term (LT-HSCs) are ring-fenced as LSK, CD150+, CD48− CD34−, and those that reconstitute short term (ST-HSCs) as LSK, CD150+, CD48−, CD34+. Nineteen percent of mouse LT-HSCs and 23% of ST-HSCs express the receptor for macrophage colony-stimulating factor (M-CSF) at their surface at a low level. The fms-like tyrosine kinase 3 (Flt3), which binds a myeloid/lymphoid affiliated cytokine, was detected at a low level on the surface of 5% of LT-HSC and 8% of ST-HSC. Flt3 expression was confirmed by the use of single-cell qRT-PCR, and 12% of LT-HSCs and 21% of ST-HSCs express mRNA for Flt3. The use of single-cell qRT-PCR has assisted in describing mouse HSC sub-sets when immunological markers are unavailable, and 13% of LT-HSCs and 19% of ST-HSCs express mRNA for the receptor for erythropoietin (Epo). Only 1% of LT-HSC and 3% of ST-HSC expressed both the receptor for Flt3 and M-CSF at their surface. The use of single-cell triplex qRT-PCR revealed that co-expression of the mRNAs for Flt3 and the Epo receptor rarely occurs [2]. Sub-sets of HSCs that selectively express the receptors for lineage-affiliated cytokine clearly exist.

For many years, an understanding of the biological activity of the hematopoietic cytokines focused on their need for colony-formation by bone marrow cells in a semi-solid medium. For example, M-CSF and erythropoietin are required for the survival and proliferation of macrophage and erythroid progenitors, respectively. From these findings, unipotent committed progenitors were seen as the population of cells that were responsive to these and other hematopoietic cytokines. Expression of the receptors for M-CSF and Epo by HSCs is highly significant because it is now known that these cytokines can instruct HSC lineage fate. The transcription factor (TF) PU.1 is myeloid associated. For *PU.1-GFP* reporter mice, intravenous injection of recombinant M-CSF increased the level of activation of *PU.1* in LT-HSCs (LSK, CD135−, CD34−, CD150+), by 16 h, and the proportion of HSCs with a myeloid lineage bias. M-CSF activation of the *PU.1* promoter was confirmed by video imaging of highly purified mouse HSCs (CD150(high)) from *PU.1-GFP* reporter mice that were treated with M-CSF in culture. The expression of PU.1 and the number of cells with a myeloid gene signature and differentiation potential were increased [3]. For granulocyte/macrophage progenitors, M-CSF commits these cells to macrophage production. Granulocyte/macrophage colony-stimulating factor and granulocyte colony-stimulating factor instruct granulocyte/macrophage progenitors to differentiate into granulocytes [4,5]. From in vivo and in vitro studies, Epo acts directly on the mouse HSC/multipotent hematopoietic progenitor cell (HPC) compartment to instruct an erythroid bias and decrease myeloid output. Gene profiling of LSK, Fl3− cells exposed to Epo in vivo revealed that genes associated with erythroid lineage commitment were up-regulated and ones that are linked to myeloid commitment were down-regulated [6].

From the instructive actions of the above cytokines on HSCs, we might expect the offspring of HSCs that express a particular cytokine receptor to perpetuate their lineage affiliation. Cell-surface markers and the use of multiplex qRT-PCR assays are limited in their ability to provide a complete description of cell subsets by virtue of the probes available to hand. To circumvent this limitation, and also provide an unbiased view on HPC heterogeneity, LSK cells (viewed as myeloid HPCs) were investigated by sequencing the mRNA of single cells by MARS-seq. The clustering of cells was based on the patterns of expression of >3461 genes and this led to the identification of 19 subsets. Seven showed transcriptional priming with various degrees of specificity towards megakaryocytes, erythrocytes, basophils, eosinophils, neutrophils, monocytes, and dendritic cells. Strikingly, there was an absence of mixed-lineage progenitors, and, therefore, lineage-affiliated HSCs perpetuate their state [7].

The above sub-typing studies revealed that some HSCs are lineage-affiliated, but did not provide proof that they have a propensity towards a particular maturation outcome. A sub-population of LSK, CD34−, CD150+ HSCs expresses the megakaryocyte-restricted surface marker CD41 (alphaIIb integrin, platelet GPIIb) and when single LSK, CD34−, CD150+, CD41+ cells were transplanted into mice they reconstituted platelets. CD34−, CD150−, CD41− cells failed to do so. Colonies comprising of CD41+ megakaryocytes were formed by culturing single LSK, CD34−, CD150+, CD41+ cells, but not by LSK, CD34−, CD150−, CD41− cells. CD41+ cells have, therefore, megakaryocyte reconstituting and colony-forming potential [8]. To investigate platelet-affiliated HSCs, other investigators generated bacterial artificial chromosome transgenic mice using a platelet marker; a *von Willbrand-eGFP* (*Vwf-eGFP*) reporter that expresses green fluorescent protein. LSK, CD34−, CD150+, CD48+ HSCs were identified that expressed *Vwf-eGFP*, and their maintenance required thrombopoietin, a regulator of platelet development. These cells are a sub-set that is primed for platelet-specific gene expression, and they showed an enhanced propensity to short- and long-term reconstitute platelets in transplantation experiments [9]. They also often showed a long-term bias towards generating myeloid cells and were able to give rise to lymphoid biased HSCs. The investigators concluded that platelet-biased HSCs reside at the apex of hematopoiesis. In this case, platelet-biased HSCs and/or their offspring HSCs are able to change their initial ‘choice’ of cell lineage. Sub-sets of mouse HSCs that are biased towards either myeloid or lymphoid development have been described within the most primitive hematopoietic cell compartment by the use of the surface markers CD150, CD42, and CD86 and exclusion of the DNA-binding dye Hoechst 33342 [10,11,12].

For human HSCs, the proportions of cells with multipotent versus unipotent lineage potential were mapped for HSC-enriched (CD34+, CD38−) and HPC-enriched (CD34+, CD38+) populations of cells from adult bone marrow by the use of an optimized single-cell assay (serum-free conditions plus a wide range of cytokines). There were few oligopotent transit HPCs in adult bone marrow, and the two classes of cells that predominated were multipotent cells and unipotent cells with a myeloid or lymphoid potential. The investigators concluded that the population structure to HPCs in adult bone marrow is two-tier. By contrast, the fetal liver contained large numbers of oligopotent progenitors, with entangled megakaryocyte, myeloid, and erythroid potentials, inferring a shift during the transition from in utero to adulthood hematopoiesis [13].

## 3. Revising the Map for Hematopoiesis

Conventional maps for the development of an entire organism, e.g., *Caenorhabditis elegans*, and a tissue, e.g., the hematopoietic cell system, are branching tree-like. The founding multipotent cells progress, via a series of binary decisions, stepwise towards oligopotent then bipotent progenitors towards a single lineage-committed cell that proliferates and differentiates to generate a substantial number of mature cells. That affiliation to a single lineage and a propensity to develop along one pathway occur as early as within the HSC compartment contradicted a branching tree-like model. In 2008, there was the need to re-write the textbook accounts of hematopoiesis [14]. At first, endeavors to re-write hematopoiesis led to a plethora of new tree-like models, and a consensus view of the conduct of hematopoiesis was lacking.

**Figure 1:**
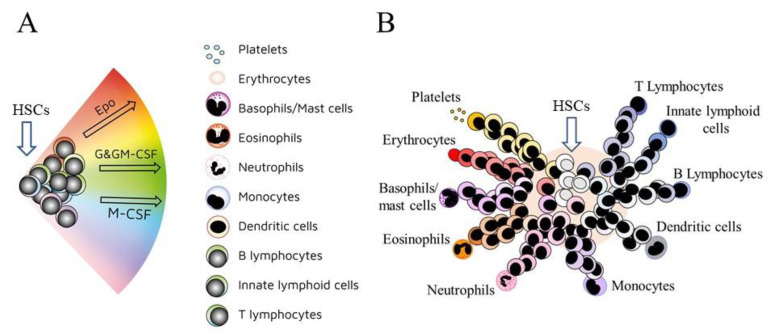
A continuum model for hematopoiesis (**A**). Hematopoietic stem cells (HSCs) ‘choose’ a lineage directly from a spectrum of the end cell options. The different colors for hematopoietic stem cells (HSCs) show that they are a mixture of cells with different lineage signatures. Erythropoietin (Epo), granulocyte colony-stimulating factor (G-CSF)/granulocyte/macrophage colony-stimulating factor (GM-CSF), and macrophage colony-stimulating factor (M-CSF) direct HSCs and hematopoietic progenitor cells towards the erythroid, neutrophil, and monocyte fates, respectively. (**B**) There are close relationships between the cell lineages, as inferred from their shared characteristics, and HSCs and hematopoietic progenitor cells retain enough versatility to ‘step sideways’ into a different pathway.

In 2009, a continuum map for hematopoiesis depicted HSCs as ‘choosing’ a cell lineage directly from a spectrum of the end cell options (Figure 1A). Even so, there are near-neighbor relationships between each of the developmental pathways, and mature cell types, as inferred from the shared use of TFs and other characteristics (Figure 1B) [15]. Complex TF circuits control hematopoiesis and TFs are differentially expressed across the hematopoietic cell states. From microarray profiling of the expression of TFs and other genes within HPCs and mature cell populations, the end cell types were placed in the near-neighbor order erythrocytes, megakaryocytes, granulocytes/monocytes, dendritic cells, B cells, natural killer cells, and T-cells, which is similar to that shown in Figure 1A [16]. 

Mouse multipotent (LSK) and oligopotent (Lin−, Sca−, Kit+) HPCs were genetically barcoded and allowed to divide in culture conditions for multi-lineage differentiation to capture the transcriptional status of cells, their developmental fate, and the relationships between pathways. Cells were sampled immediately and later for single-cell RNA sequencing to allow the clonal tracing of transcriptomes. The nine cell types that appeared in culture were megakaryocytes, erythrocytes, basophils, mast cells, eosinophils, neutrophils, monocytes, dendritic cells, and lymphoid precursors. Findings for the least differentiated HPCs provided strong support to the notion that HSCs and HPCs lie along a continuum of transcriptional states, rather than matching to a discrete hierarchy of oligopotent and intermediate HPCs. Uni-lineage differentiation was exhibited by some clones, and multilineage by others. A map was constructed for the transcriptional landscapes of the cells that appeared after 6 days in culture to examine how multipotent HPCs veered towards pathways. The observed order of near-neighbors was megakaryocytes, erythrocytes, mast cells, basophils, eosinophils, neutrophils, monocytes, migratory dendritic cells, plasmacytoid dendritic cells, and lymphoid precursors, as for the map in Figure 1B. Cells that are mostly LT-HSCs and ST-HSCs (Lin−, Sci(high), Kit+) were barcoded, cultured for two days, and transplanted into irradiated mice. Analysis of the transcriptomes of the cells that were recovered post-transplantation also revealed a continuum landscape for hematopoiesis from multipotent cells with pathways, as to near-neighbors, towards erythrocytes, basophils, neutrophils, monocytes, dendritic cells, B cells, and T cells [17].

Evidence that lineage commitment is a continuous process for human HSCs and their immediate progeny (Lin−, CD34+, CD38-) and more differentiated HPCs (Lin−, CD34+, CD38+) was obtained by constructing developmental trajectories from combining the findings from single-cell RNA sequencing and single-cell culture differentiation outcomes. The more mature Lin−, CD34+, CD38+ cells separated into clusters that conformed to distinct HPCs for each of the major hematopoietic cell types. There was an absence of clusters for Lin−, CD34+, CD38- cells, revealing that these cells were a single and continuously connected entity. From more detailed analysis, the investigators concluded that low-primed HSCs/HPCs (termed CLOUD hematopoietic stem and progenitors) gradually acquire continuous lineage priming in multiple directions towards the major pathways of development. Similarly, cell sub-populations that had been ringfenced and sorted as various oligopotent progenitors, by virtue of their surface marker expression, were mainly cell types with single lineage gene expression and functional lineage potency. A graphical summary of a continuum model placed near-neighbor cell lineages as erythrocytes, megakaryocytes, eosinophils/basophils/mast cells, neutrophils, monocytes/dendritic cells, and B cells, which is similar to that shown in Figure 1B [18].

Lineage-affiliated HSCs and HPCs remain versatile because they can still adopt a pathway that is different from their initial choice. Thymocyte progenitors can give rise to macrophages, dendritic cells, and natural killer cells. The earliest T cell progenitors possess macrophage fate, M-CSF was required for macrophages, and as above instructs macrophage fate [19,20]. Findings from RNA sequencing of more than 1600 single mouse HSCs and HPCs and then constructing expression maps revealed that the trajectories are broad for cells that are developing along the erythroid, neutrophil/macrophage, and lymphoid pathways. The investigators argued that developing cells also have the option to move to the left or right of a chosen pathway [21]. Other workers have proposed from their analysis of the lineage status of mouse HPCs that bursts of alternative gene expression underlie bi-lineage states that are flexible. For example, Gfi1 and lrf8 are expressed at a low level in cells that are veering towards neutrophils/macrophages and there is selective increased expression of Gfi1 and lrf8 during neutrophil and macrophage development, respectively [22]. The megakaryocyte and erythroid trajectories share a dependence on the TF GATA-1, and megakaryocyte-primed human HSCs can step sideways towards erythropoiesis [23]. A range of related cell types (see Figure 1B) are observed in the colonies when bone marrow cells are dispersed in a semi-solid medium. The plated cells are devoid of their normal social environment and the dividing cells may, therefore, be stepping sideways.

An intriguing question is where might the control to HSC versatility lie, particularly regarding the ability of cytokines to direct a cell’s lineage fate and that modifications to the epigenome are subject to external influence. As early as 1957, Waddington proposed a theoretical landscape of epigenetic hills and valleys that restricts the pathways of developing stem cells. This model has provided a metaphor that is still used to describe bifurcations during developmental processes [24]. CpG sites throughout the genome (the methylome) have been examined for multipotent HSCs as they develop along different pathways. The cells examined were sorted according to their surface phenotype, and, as such, designated as mouse multipotent, myeloid, lymphoid, granulocyte/macrophage, and thymocyte progenitors. As above, the cells within the oligopotent HPC populations may well have single lineage gene expression. Albeit, sets of genes were progressively and differentially hypermethylated, and silenced, as cells veered towards myeloid versus lymphoid fates. Upon myeloid progression of multipotent HPCs, differentially methylated regions that were hypermethylated substantially exceeded ones that were hypomethylated. When myeloid HPCs veered towards becoming granulocyte/macrophage HPCs, nearly all of the differentially methylated regions showed loss of methylation. From the above, the key findings are that genes are progressively hypermethylated and that there is dynamic plasticity in methylation as HPCs develop [25]. Intriguingly, induced pluripotent stem cells generated by direct transcription-factor-based reprogramming of somatic cells retain an ‘epigenetic memory’ of their tissue cell of origin. Whilst this might reflect incomplete reprogramming another possibility is that developmental programming still allows cells to retain plasticity regarding the adoption of other options [26].

The existence of lineage-affiliated and differentiation-biased HSCs has blurred a longstanding demarcation between self-renewing HSCs and transit HPCs that were viewed as an amplifying population that progressively ‘age’ to give rise to mature cells. Instead, self-renewing cells are twofold, namely ‘primary’ multipotent and ‘secondary’ lineage-affiliated/biased HSCs. It is noteworthy that unbiased and global analyses of cell status have provided strong support to a continuum model. By contrast, the ring-fencing of HPCs, with regard to surface phenotype and the use of cytokines to grow colonies of cells in a semi-solid medium consisting of particular cell types, appears now to have misled mapping of hematopoiesis by virtue of their inherent selectively. To some extent, the monophyletic and dualistic views on HSCs were both close to the mark. There are two possibilities regarding lineage-affiliated/biased HSCs veering towards ultimately generating an abundance of one type of mature cell. There might be a discrete/stepwise transition to a unipotent HPC or the process is gradual and continuous [27]. Perhaps, the inherent versatility of HSCs and HPCs excludes a discrete step that truly commits cells to one pathway. There are near-neighbor relationships between the routes to the end cell types and the end cells per se, but there is still the need for a consensus on the precise ordering of cell relationships.

## 4. The Target Cells for Leukemia

From the early 1980s, a ‘classic’ tree-like map for the process of hematopoiesis was used to map the origins of the leukemias. The three-tier hierarchy of cells included self-maintaining HSCs, various aging HPCs that have stepwise limited potentials, and mature cells that eventually die. HPCs that were committed to a single cell lineage were viewed as ‘targets’ for some leukemias and provided a simple rationale as to why the various differentiated or partially differentiated leukemia and lymphoma cells belong to just one cell lineage. For example, B cell-committed progenitors were designated as giving rise to childhood acute lymphoblastic leukemia (ALL) that is non-B and -T cell [28]. Some mature subsets of B and T lymphocytes were viewed as ‘targets’ due to their proliferative potential and long life span. These cell populations were viewed as giving rise to, for example, B-non-Hodgkin lymphoma, B-chronic lymphocytic leukemia, T-chronic lymphocytic leukemia, and T-cutaneous lymphoma.

However, there are two conceptual difficulties to the notion that unipotent HPCs are prime ‘targets’ for transformation. The time scale for the development of leukemias is several years in man and several months in mice. The transformed cell needs to stay in the body for a least a few years or months for leukemia to develop and be sustained. By comparison, the time scale for the transit of an erythroid- or granulocyte-committed progenitor in generating mature cells, resulting in the exhaustion of these cells, is much shorter, and for mice around 15 days. A further confounding issue to the matter of time scale relates to identical twins that both develop childhood ALL. At least two genomic insults are required to develop this disease. Occurrence in both twins is due to the intra-placental sharing of a pre-leukemic clone with the same chromosomal translocation, and asynchronous with a latency that is variable and occasionally very protracted. In this case, the initial ‘targeted’ cell, termed a leukemia-initiating cell, has remained ‘dormant’ for quite some time. A second postnatal insult(s) converts the initiating cell into a leukemia stem cell (LSC) for disease [29]. This adds a further ‘latency’ to the time scale for leukemia development. A second conceptual difficulty to viewing a unipotent HPC as a ‘target’ is that there are no clear findings to support the view that an oncogene can endow a committed progenitor, or a mature cell subset, with the capacity to self-renew for cancer, as brought to attention for the origins of the malignant squamous cell carcinomas [30].

### 4.1. Myeloid Leukemias

It has been known for some time that human chronic myeloid leukemia (CML) is a clonal disease that arises in a transformed (Philadelphia chromosome-positive (Ph+)) multipotent HSC [31]. The end cell product of this clone is a substantial rise in the number of granulocytes that have largely differentiated normally. Bactericidal activity of the granulocytes is not impaired, but their chemotactic activity is reduced [32]. Why is the production of leukemia cells in CML restricted to granulocytes? A possibility is that this is safe and carries the least risk to the organism from the disease. However, this is somewhat an anthropomorphic view, and how might control of hematopoiesis pay regard to ‘safety’. Another view is that the generation of granulocytes is an inherent property of the CML LCSs, as provoked by the oncogenic insult. Human acute erythroleukemia (acute myeloblastic leukemia FAB-M6) also originates in an HSC and there is an accumulation of erythroblasts and/or myeloblasts. The disease subsets are myeloblast-rich (M6A), pro-erythroblast-rich (M6B), and myeloblast- and pro-erythroblast-rich (M6C). Like CML and for M6A and M6C, LSCs are directed to generate one type of cell [33].

### 4.2. Acute Lymphoid Leukemias

B-cell precursor ALL in infants (<1 year of age) is an aggressive disease and ~80% of cases have chromosomal rearrangement resulting in the *MLL-AF4* fusion gene. B-cell precursor ALL has a neonatal origin as revealed by twin concordance studies and the analysis of Guthrie cards and the identification of gene fusion sequences in neonatal blood spots [34]. There is an accumulation of pro-B cells in the bone marrow. A multilayered genome-wide analysis of 124 de novo cases revealed transcriptional similarities between the leukemia cells and the most immature human fetal liver HSCs and HPCs. From RNA sequencing, the gene expression sequence for B-cell precursor ALL cells resembled Lin−, CD34−, CD38−, CD19− fetal liver cells. This population of cells includes HSCs, multipotent HPCs, and lymphoid-primed multipotent progenitors (LMPP). All of these populations lie upstream of fetal liver B cell progenitors which are CD34+, CD19+ [35]. A mouse model of B-cell precursor ALL was developed by targeting *MLL-AF4* to HSCs during embryonic development. The model did not recapitulate the aggressive human leukemia. Hence, the hematopoietic compartment was separated into HSCs/multipotent HPCs, LMPP, and lymphoid progenitors, and gene expression analysis and transplantation assays were used to identify the cell-of-origin of B-cell precursor ALL. MLL-AF4+ led to an enhanced engraftment of just the LMPP fraction and a strong B cell bias was seen in the recipient mice. Ikarus and Bcl-2 were upregulated in the MLL-AF4+ LMPPs. From these findings, the investigators concluded that fetal liver LMPPs provide the prerequisites for the initiation of B-cell precursor ALL [36]. Even so and bearing in mind that HSCs and HPCs are versatile, it is interesting that, in addition to the maturation arrest in B-cell precursor ALL, the cells are restricted to the B cell pathway. It is important to bear in mind that LMPPs are lympho-myeloid stem cells; they still have neutrophil, monocyte, B cell, and T cell potentials but fail to produce significant erythroid and megakaryocyte offspring [37].

Further studies have revealed a possible HSC origin for some leukemias that were viewed as arising in a lineage-committed progenitor. Around 75% of cases of childhood ALL belong to the B-cell precursor subtype, and the remainder carry T-cell progenitor markers [38]. As above, the B-cell precursor subtype has been thought to arise in a B cell committed progenitor [28], and ~97–99% of the leukemia cells express the early stage B cell antigen CD19. The identification of a very minor fraction of leukemia marrow cells that have a primitive Lin−, CD34+, CD19−, CD33−, CD38− phenotype and that contained the patient-specific leukemia karyotype suggests that B-cell precursor ALL may arise in a more primitive cell [39]. Bromodeoxyuridine labeling examination of the proliferative status of bone marrow cells from 15 children with the B-cell precursor subtype prior to treatment identified a small population of primitive blast cells that were CD19− and nonproliferating. CD19- cells were sorted and stained for CD10 or CD34, and the CD19−, CD10− and C19−, CD34− cells were almost nonproliferating. CD19− cells were proven to be leukemic by performing FISH analysis for two ETV6/RUNX1 positive cases [40]. Similarly, to replicate human ALL in mice required HSC-like cells (CD34+, CD10− or CD34+, CD19−) that lacked B cell markers [41]. The above primitive cells may be the LSCs in childhood ALL and are restricted to B cell development despite being upstream of B-cell progenitors. However, there is still debate about whether the LSCs in the B-cell precursor subtype ALL arise from the transformation of an HSC or from a more committed progenitor that has acquired a stem cell-like nature [42].

### 4.3. Acute Promyelocytic Leukemia (APL)

Acute promyelocytic leukemia is the most differentiated form of acute myeloid leukemia (AML-M3). The promyelocyte is an unlikely target for transformation because this cell has limited proliferative potential. A longstanding notion is that APL stems from a myeloid progenitor, but the evidence for such is conflicting [43]. The hallmark of APL is the t(15;17) translocation which generates the PML-RARα oncoprotein [44]. This fusion protein has been viewed as inhibiting the differentiation and promoting the survival of myeloid progenitors [45]. PML-RARα expression in the CD34+, CD38+ cells, but not in the more primitive CD34+, CD38− cells, from three patients, supported the case for a myeloid committed progenitor as the origin of APL [46]. However, a later study showed, by sorting cells and in situ hybridization, that the t(15;17) translocation is present in both the CD34+, CD38− and CD34+, CD38+ cells from patients, suggesting that there is transformation of a cell within the more primitive CD34+, CD38− cell compartment and, therefore, there is lineage restriction of the leukemia cells [47].

### 4.4. B Cell Leukemias and Lymphomas

Chronic lymphocytic leukemia presents as a malignancy of small B cells. However, there is an abnormal expression of lymphoid genes in patients’ HSCs which when purified produce a high number of polyclonal B-cell progenitors. Upon xenogeneic transplantation, CLL-HSCs gave rise to clonal B-cells. These findings indicate that the propensity to a clonal B cell malignancy is set at the HSC stage [48]. This was a surprising finding, with major implications for the treatment of CLL [49]. By contrast, many B cell lymphomas are viewed as arising from germinal center B cells because of their high rate of proliferation and a highly active mutagenic process [50].

From the need for the cells that give rise to leukemia to have the capacity to self-renew and a long life span, HSCs are primarily ‘at risk’ to malignant transformation. In addition, some leukemias are highly unlikely to arise in a committed HPC or a mature cell type that is unable to sustain itself in the body for years. Indeed, and from studies of acute myeloid leukemia (AML), the stem cell theory of cancer postulates that most, if not all, cancers arise from a normal stem cell that gives rise to the cell types of a specific tissue [51]. LSCs generate a hierarchy of cells [51,52], and for the above human leukemias the offspring of LSCs, unlike those of HSCs, belong to just one cell lineage.

The search for the cells that maintain a cancer has been extended to many other cancers, and the proportion of LSCs/cancer stem cells (CSCs) ranges from very few (0.1–0.0001%) to 27% [53,54,55]. Hence, the hierarchy of cells within a cancer varies for the different types of cancers. Additionally, there is still uncertainty about the precise nature of the cell-of-origin for many solid cancers. For example, brain tumors are very heterogeneous as they arise from multiple types of cells regarding their location within the nervous system. These tumors contain a minor population of cells that are neural or glial stem cell-like and these cells and progenitor-like cells are more readily transformed by the activation of oncogenes than differentiated neuronal cells. Whilst transformed neural and glial stem cell-like cells are thought to maintain brain tumors, fate-restricted cells cannot be excluded. Regarding a possible stem cell origin, there are findings to indicate that developmental processes are altered in brain tumors so that the malignant cells are ‘locked into’ a program reviewed in [56].

## 5. Targeting of Oncogenes to Hematopoietic Stem Cells

Oncogenes are well known to promote the avoidance of apoptosis [57] and cell proliferation [58]. HSCs are the origin of some leukemias, and, therefore, might some oncogenes influence the developmental fate of HSCs to dysregulate tissue homeostasis. The possibility of this disruption was examined by restricting the expression of some oncogenes to HSCs/HPCs in transgenic mice and examining the cell lineage of the leukemia cells. Oncogenic events that have been targeted to HSCs/HPCs and their specific and consistent association with a particular type of leukemia are shown in Table 1.

### 5.1. The BCR-ABLp210 and BCR-ABLp190 Fusion Genes

**Figure 2:**
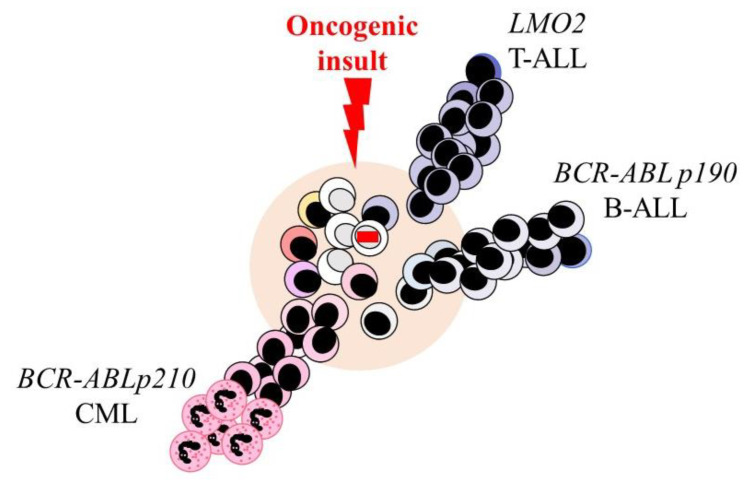
Targeting of *BCR-ABLp210*, *BCR-ABLp190*, and *LMO2* to hematopoietic stem and progenitor cells in transgenic mice leads to the development of myeloid, B-cell, and T-cell leukemia. Expression of these oncogenes was restricted by mean of the stem cell antigen 1 (Sca1) promoter.

The reciprocal t(9;22) chromosomal translocation in human CML fuses the *BCR* gene to the *ABL* proto-oncogene [59,60] to generate the BCR-ABLp210 oncoprotein. As mentioned above for human CML, the offspring of the transformed HSCs are granulocytes. BCR-ABLp210 expression was restricted to the HSC/HPC compartment in transgenic mice by means of stem cell antigen 1 (Sca1)-BCR-ABLp210. This led to a myeloid leukemia that recapitulated the human disease (Figure 2) [61,62]. DNA methyltransferase 1 was upregulated within the HSCs/HPCs from the Sca1-BCR-ABLp210 mice and Sca1-Dnmt1 transgenic mice developed a malignancy whereby granulocytes were expanded in the blood and bone marrow. Hence, Dnmt1 provides a link between BCR-ABLp210 expression and the epigenetic programming of HSCs/HPCs towards granulocytes [62]. BCR-ABLp210 is clearly not important to the survival of the leukemic granulocytes as it is not expressed by these cells, by virtue of Sca1 control of BCR-ABLp210. Nor is the ABL tyrosine kinase activity needed for the survival of the transformed HSCs/HPCs. CML The reason is that LSCs persist post the use of kinase inhibitors to treat CML as revealed by disease relapse post-cessation of treatment [63]. It seems that the role of BCR-ABLp210 is to ‘set’ the lineage of HSCs/HPCs and their offspring to granulocytes.

Cells that have the fusion gene *BCR-ABLp190* are present in neonatal cord blood and the blood of healthy adults, but the majority of carriers do not develop Ph+ precursor B-cell ALL [64,65,66]. Therefore, whilst *BCR-ABLp190* might promote leukemogenesis a second hit is needed. To investigate the role of *BCR-ABLp190* in leukemogenesis investigators targeted the pre-leukemic oncogenic lesion to HSCs/HPCs in transgenic mice by means of *Sca1-BCR-ABLp190*. This resulted in precursor B-cell ALL resembling the human disease, but the penetrance was low (13%) in an aging mouse colony. By contrast, precursor B-cell ALL developed with a 90% incidence and much shorter latency in transgenic mice that were double *Sca1-BCR-ABLp190* and *Pax5* (a B cell TF) +/−. Genomic alterations also accumulated in the remaining wild-type *Pax5* allele. The investigators concluded that loss of *Pax5* together with *BCR-ABLp190* confers changes that are essential for precursor B-cell ALL whereby *Sca1-BCR-ABLp190* has set the cell lineage of HSCs/HPCs. Regarding the role of *Pax5*, pro-B cells from *Pax5* deficient mice had increased glucose uptake and energy metabolism, with upregulation of metabolic genes, as seen for human leukemia [67].

### 5.2. The ETV6-RUNX1 Fusion Gene

The most common acquired fusion gene in childhood B-ALL is *ETV6-RUNX1* (known also as *TEL/AML1*), accounting for 25% of cases [68]. This fusion gene confers a low risk of developing the disease because pre-leukemic clones are found in neonatal blood with few carriers developing the disease. An abnormal reaction to a common infection, triggers critical secondary mutations, has been proposed as a causal mechanism for the onset of childhood ALL [69]. This need for two oncogenic insults for childhood B-ALL has been investigated by targeting *ETV6-RUNX1* expression to various hematopoietic cell compartments. When *ETV6-RUNX1* expression was targeted to the B cell lineage (at the pro-B cell level) none of the mice developed leukemia even upon exposure to natural infections. By contrast, infection exposure triggered leukemogenesis when *ETV6-RUNX1* expression was initiated in HSCs/HPCs. The leukemias that developed included both T-cell (35%) and B-cell (6%) ALL, and notably no myeloid malignancies. The *KDM5C* gene encodes H3K4me3 and H3K4me2 demethylase. *KDM5C* is missense mutated in mouse *ETV6-RUNX1* B-ALL and human relapse *ETV6-RUNX1* B-ALL [70]. Introduction of *KDM5C* loss into the B cell compartment of mice that expressed *ETV6-RUNX1* did not give rise to B-cell ALL. Hence, loss-of-function of KDM5C at the B-cell stage does not lead to the preleukemic clone that expresses ETV6-RUNX1 giving rise to leukemia. By contrast, the introduction of *KDM5C* loss-of-function in HSCs/HPCs for mice that expressed *ETV6-RUNX1* led to 22% of the mice developing B-ALL when kept in a special pathogen-free environment. From these findings, and gene expression profile studies, the investigators concluded that *ETV6-RUNX1* can trigger T-cell and B-cell leukemias, the second ‘hit’ determines the leukemia cells’ lineage identity, and both ‘hits’ have to occur in an HSC/early HPC [71].

### 5.3. The Active LMO2 Gene

Chromosomal translocations that active the *LMO2* gene are exclusive to T-cell leukemias [72]. Targeting expression of *LMO2*, in transgenic mice via the Sca1 promoter, led to a human-like T-ALL that was highly disseminated. Additionally, targeting expression to cells at the pro-B cell stage of development also led to the development of an aggressive T-ALL. Transgenic mice also developed an aggressive T-ALL when the expression of *LMO2* was targeted to germinal center B-cells. LMO2 can, therefore, program different cell populations to set the identity of the target cell and their offspring to a T-cell leukemia identity [73].

As outlined above, some oncogenes can set the cell lineage of HSCs/HPCs and their offspring. Transformation of lineage-affiliated HSCs might well offer an immediate explanation of why the leukemia cells belong to just one cell lineage. However, two findings favor the view that the oncogene per se programs the fate of HSCs/HPCs. For *ETV6-RUNX1* transformation, the outcome is B-ALL and the cell transformed is developmentally prior to the pro-B cell stage. For *LMO2* transformation, the outcome is T-ALL and *LMO2* reprograms the pro-B cell stage of development and germinal center B cells leading to T-cell ALL. Regardless of whether a multipotent or lineage-affiliated HSC is transformed, normal HSCs and HPCs remain versatile. As outlined above, they are still able to veer towards another cell lineage, whereas the offspring of LSCs appear to be ‘fixed’ regarding their cell lineage.

### 5.4. Epigenetic Changes

The findings from studies of *BCR-ABLp210* transformation, which led to CML in the transgenic mice, indicate that the action of the oncogenes in setting the lineage fate of the leukemia cells is by virtue of epigenetic reprogramming. There are changes to the epigenome in leukemia, and other cancers, and these changes are inheritable is well described. The changes include modifications to DNA, without changing its sequence, and to histones, and dysregulation of the expression of miRNAs. Examples for myeloid disorders include overexpression of Dnmt1 in AML and myelodysplastic syndromes [74], inactivating mutations of the histone methyltransferase gene *EZH2* in myeloid disorders [75], and downregulation of the tumor-suppressor mRNA-495 in MLL-rearranged AML [76]. It is perhaps not too surprising that the epigenome is targeted by oncogenes that disrupt the lineage fate of a stem cell because the epigenome has been described as “the judge, jury, and executioner” to stem cell development [77]. Epigenetic changes are reversible and agents that erase epigenomic ‘marks’ have been explored as potential therapeutics for cancer. The histone deacetylase inhibitor valproic acid is cytotoxic against AML blast cells in vitro [78] and has been used with *all*-trans retinoic acid and intensive chemotherapy for older AML patients. However, the addition of valproic acid did not improve the complete remission rate and patients achieving a remission still relapsed [79]. It seems that the oncogene-provoked aberrant epigenetic state of LSCs is by no means readily reversible and that the cell lineage of LSCs is fixed in an obstinate manner.

## 6. Implications to the Treatment of Leukemia

There is unequivocal proof that LSCs exist in CML that arise from the transformation of an HSC. Rare and quiescent LSCs were isolated from patients with CML, and they were found to be insensitive to high doses of chemotherapeutic agents that target cells that are cycling [80]. CML-LSCs are also resistant to the tyrosine kinase inhibitors, such as imatinib, that are used to treat CML and other cancers [63,81]. Accordingly, finding a cure for CML is still a challenge, particularly as residual CML-LSCs largely cause disease relapse. The difficulty in eradicating CSCs is by no means limited to CML. For example, lung cancer is the most common cancer globally and lung cancer stem cells have gained increasing attention [82]. They are characterized by the use of a panel of markers (CD133+, CD34+, ABCG2+, ALDHIA1+), form spheroids, and give rise to colonies in vitro. Whilst the cell-of-origin of lung cancer CSCs is still under question, the expression of CSC markers is associated with resistance to anticancer therapies. Therefore, lung cancer CSCs are considered as key targets for therapies as lung cancer treatment is confounded by relapse post-treatment. To develop novel therapeutic approaches, investigators have examined the role of a number of signaling pathways, for example, phosphatidylinositol 3-kinase/Akt and Hedgehog, and targeting the niche that the CSCs reside in.

The need is to focus on identifying the attributes of normal stem cells that are aberrant/overexpressed in CSCs and crucial to their survival, with a view to achieving selective killing of CSCs. The retinoic acid receptors (RAR) RARα, RARβ, and RARγ play key roles in embryonic and adult cell development and exemplify the need to focus on stem cell attributes because, as from studies of hematopoiesis, expression of RARγ is restricted to HSCs and their immediate offspring. Accordingly, RARγ plays a role in the maintenance of HSCs [83]. Importantly, RARγ is a putative oncogene for a number of cancers. Some AML patients’ cells have RARγ fusion proteins. RARγ is often over-expressed in colorectal and renal cancer and RARγ promotes the growth of hepatocellular cancer xenografts in mice reviewed in [84]. Prostate cancer cells depend on RARγ activation for their survival; the use of a synthetic retinoid to antagonize RARγ kills prostate cancer CSC-like cells [85]. Antagonizing all RARs kills breast cancer CSC-like cells and the CSCs that give rise to neurosphere-like structures from pediatric patients’ primitive neuroectodermal tumors and astrocytoma [84]. The RARγ antagonist is, therefore, a promising new therapeutic for the above cancers. Even so, achieving sufficient selectively is a longstanding paradigm to the development of new cancer treatments, and perhaps more germane regarding the need to eradicate CSCs and spare normal stem cells.

## 7. Concluding Remarks

New findings that are changing conventional wisdom regarding the cells that are ‘targets’ for oncogene transformation are clearly highly important to the tactics for developing better treatments for cancer. Current treatments, including conventional chemotherapy and radiotherapy, are directed against the bulk of the cancer cells that are proliferating. The response is dramatic for some cancers, but many current treatments are unlikely to cure cancer and/or result in long-term remissions by virtue of failing to eliminate quiescent and residual LSCs/CSCs [86]. These cells may often arise from oncogene transformation of a multipotent tissue-specific stem cell with the offspring being restricted to one type, and there is the need to develop treatments that kill LSCs/CSCs. Developing therapy strategies that take into account the concept of CSCs and how these cells differ from normal stem cells would revolutionize how we treat many cancers.

## Figures and Tables

**Table 1:** The specific leukemia associations of the oncogenes targeted to HSCs/HPCs.

Oncogenic Event	Leukemia Association
The *BCR-ABLp210* fusion gene	CML
The *BCR-ABLp190* fusion gene	Ph+ precursor B-cell ALL
The *ETV6-RUNX1* fusion gene	Childhood B-ALL
Chromosomal translocation activation of the *LMO2* gene	T-cell leukemia

## Data Availability

Not applicable.
